# Two chromosome-level genome assemblies of galling aphids *Slavum lentiscoides* and *Chaetogeoica ovagalla*

**DOI:** 10.1038/s41597-024-03653-x

**Published:** 2024-07-20

**Authors:** Shifen Xu, Liyun Jiang, Zhengting Zou, Ming Zou, Gexia Qiao, Jing Chen

**Affiliations:** 1 Key Laboratory of Zoological Systematics and Evolution, Institute of Zoology, Chinese Academy of Sciences, Beijing, China; 2 College of Life Sciences, University of Chinese Academy of Sciences, Beijing, China

**Keywords:** Genome, Entomology

## Abstract

*Slavum lentiscoides* and *Chaetogeoica ovagalla* are two aphid species from the subtribe Fordina of Fordini within the subfamily Eriosomatinae, and they produce galls on their primary host plants *Pistacia*. We assembled chromosome-level genomes of these two species using Nanopore long-read sequencing and Hi-C technology. A 332 Mb genome assembly of *S. lentiscoides* with a scaffold N50 of 19.77 Mb, including 11,747 genes, and a 289 Mb genome assembly of *C. ovagalla* with a scaffold N50 of 11.85 Mb, containing 14,492 genes, were obtained. The Benchmarking Universal Single-Copy Orthologs (BUSCO) benchmark of the two genome assemblies reached 93.7% (91.9% single-copy) and 97.0% (95.3% single-copy), respectively. The high-quality genome assemblies in our study provide valuable resources for future genomic research of galling aphids.

## Background & Summary

Some insects can induce abnormal growth and development of host plants and form highly specialized structures termed galls. Galls benefit insect inducers and their offspring by providing abundant nutrition and protection against natural enemies and adverse abiotic factors^1^. Gall formation at the early stage is generally induced by insect stimuli, including feeding and oviposition^1^. The molecular mechanisms of gall induction and development have been found to be inseparable from insect effectors^2,3^ and phytohormones^4–6^. Aphids (Hemiptera: Aphidoidea) are an important group of plant-sapping insects that comprise over 5,000 species, 10–20% of which can induce galls on their primary host plants^7,8^. Galling aphid species mainly belong to Adelgidae, Phylloxeridae, and several subfamilies of Aphididae, including Eriosomatinae, Hormaphidinae, Tamaliinae, Thelaxinae, and Aphidinae^9,10^. To date, reference genomes are available for more than 40 aphid species. Among them, only seven true gall-forming species have been sequenced and assembled^11–16^. The lack of genomic resources for galling aphids has greatly hindered our understanding of the genetic basis for adaptive evolution of gall induction in aphids.

The tribe Fordini of subfamily Eriosomatinae is a typical lineage of true gall-inducing aphids. Species from one subtribe, Melaphidina, induce galls on *Rhus* (Anacardiaceae). The *Rhus* galls usually contain high concentrations of tannins and have economic and medicinal values^17^. The genome of one representative species, *Schlechtendalia chinensis*, has been reported recently^15^. Aphids within the other subtribe, Fordina, induce galls on *Pistacia* (Anacardiaceae), which is an economically significant genus of plants. *Pistacia vera* (cultivated pistachio) produces edible nuts that are of great commercial importance. Seeds of other wild *Pistacia* species also possess economic value and can be utilized for local consumption, oil extraction, soap production, and most importantly, as rootstock sources for pistachio trees^18^. Extracts from *Pistacia* galls exhibit anti-inflammatory and antioxidant activities^19,20^. However, no genomic data are available for this galling group.

**Fig. 1:**
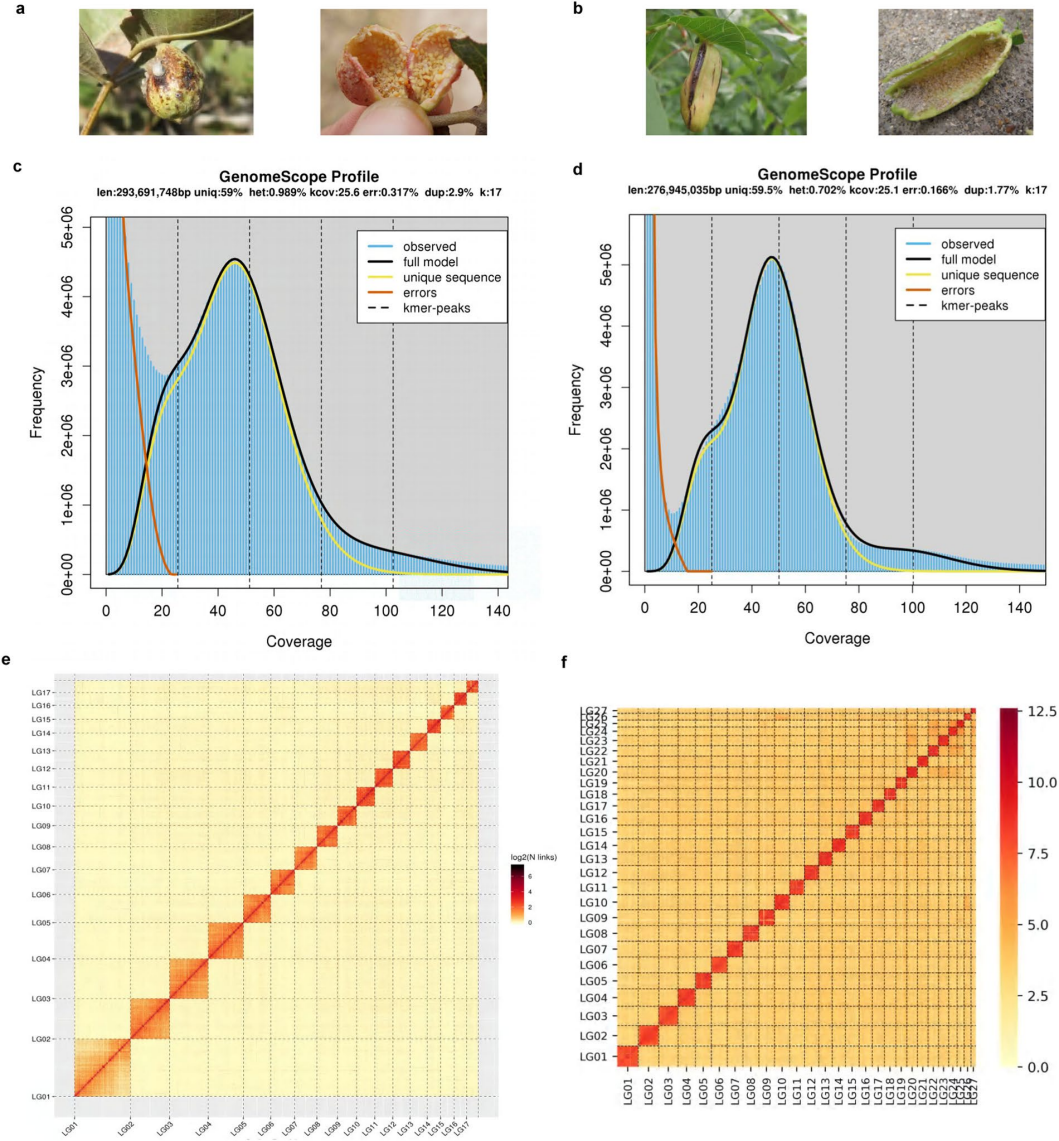
Aphid galls and genome features of *Slavum lentiscoides* and *Chaetogeoica ovagalla*. Galls and aphids living in galls of *S. lentiscoides* (**a**) and *C. ovagalla* (**b**). K-mer distribution plots for genomes of *S. lentiscoides* (**c**) and *C. ovagalla* (**d**). Hi-C interaction heatmaps for genomes of *S. lentiscoides* (**e**) and *C. ovagalla* (**f**).

Producing more high-quality genome assemblies encompassing different lineages is foundational to the genomic study of galling aphids. In this study, we generated chromosome-level genome assemblies of two galling species from Fordina, *Slavum lentiscoides* Mordvilko and *Chaetogeoica ovagalla* (Zhang). Both species induce closed bag-like galls on the main veins of *Pistacia* spp. leaves^8^ (Fig. 1a,b). A total of 332.26 Mb and 289.72 Mb assembled sequences were anchored to 17 and 27 pseudo-chromosomes for *S. lentiscoides* and *C. ovagalla*, with 11,747 and 14,492 predicted protein-coding genes, respectively.

## Methods

### Sample collection

*S. lentiscoides* samples within galls were collected on a pistachio tree (*P. vera*) from Andijan, Uzbekistan (40.719°N, 72.436°E) in September 2019. The galls of *C. ovagalla* were obtained on *Pistacia chinensis* from Qingdao, China (36.193°N, 120.573°E) in July 2018. The aphids from fresh galls were rapidly frozen and stored in liquid nitrogen at the National Animal Collection Resource Center, Institute of Zoology, Chinese Academy of Sciences, Beijing, China.

### Genome sequencing

Total genomic DNA was extracted from over 0.2 grams of aphids within a single gall using a standard CTAB method. For Oxford Nanopore sequencing, the concentration and quality of DNA were checked using 1% agarose gel electrophoresis, NanoDrop spectrophotometry (Thermo Fisher Scientific, Lafayette, USA), and Qubit fluorometry (Invitrogen, Darmstadt, Germany). Large-sized segment libraries were selected (≥30 kb) with the BluePippin^TM^ System and processed by the ONT Template Prep Kit (SQK-LSK109, Oxford Nanopore Technologies [ONT], Oxford, UK) protocol. DNA fragments were end-repaired and 3′-adenylated using the NEB Next FFPE DNA Repair Mix kit (New England Biolabs [NEB], Ipswich, USA). The Nanopore sequencing adapters were ligated using the NEBNext Quick Ligation Module (E6056) (NEB). The final library was sequenced on R9 flow cells using a PromethION DNA sequencer (ONT) with an ONT sequencing reagent kit (EXP-FLP001.PRO.6). The raw signal data were called, and the FAST5 files were converted into FASTQ files using MinKNOW software v2.0 (ONT). Short reads (<2kb) and reads with low-quality bases and adapter sequences were removed. Shotgun sequencing reads were used to estimate genome size and to correct the genome assembly. Paired-end libraries were prepared following the instructions for sequencing with the NovaSeq 6000 Reagent Kit on a NovaSeq 6000 platform, with an insert size of 350 bp. For Hi-C sequencing, nuclear DNA from tissue cells was cross-linked and enzymatically digested with DpnII. The DNA fragments with interaction relationships were captured with streptavidin beads and prepared for sequencing. Hi-C libraries with 300–700 bp insert sizes were constructed on the PE150 Illumina platform. Total RNA was extracted from the same colony for RNA extraction and sequencing. RNA-seq libraries were constructed using the NEBNext Ultra RNA Library Prep Kit for Illumina (NEB) and were then sequenced on the NovaSeq 6000 platform with a 150-bp paired-end output.

We finally generated for the first time chromosome-level genome assemblies of *S. lentiscoides* and *C. ovagalla* using a combination of Nanopore long reads (44.14 Gb and 55.02 Gb, respectively), Illumina short reads (58.13 Gb and 36.40 Gb, respectively), and Hi-C sequencing data (40.35 Gb and 45.54 Gb, respectively).

### Karyotype analysis

We dissected young embryos of aphids on slides. The embryos were kept in 0.7% sodium citrate for 30 min and then fixed in Carnoy’s fixative for 15 min. After fixation, a coverslip was placed on the slide and vertically pressed down to disperse the cell mass as much as possible. The treated slide was frozen at −80 °C for 10 min, followed by air drying. The air-dried slide was stained in 5% Giemsa solution for 15 min and then washed with a tiny stream of distilled water. The slide was observed and subjected to photomicrography using a Leica DM6B photomicroscope (camera magnification 100 × 10). The result showed that *S. lentiscoides* has a diploid chromosome number of 2n = 34 (Fig. S1), while the karyotype of *C. ovagalla* was not obtained due to sample limitations.

### Genome assembly

Before genome assembly, we extracted Illumina paired sequencing reads with approximately 50× coverage to estimate genome size and heterozygosity. The 17 k-mer frequency spectra obtained with Jellyfish v1.1.10^21^ and GenomeScope^22^ suggested that the heterozygosity of the *S. lentiscoides* and *C. ovagalla* genomes was 0.99% and 0.70%, the estimated genome sizes were 293.69 Mb and 276.95 Mb, and the repeat sequence content was 41.0% and 40.5%, respectively (Table 1, Fig. 1c,d).

**Table 1:** Genome assemblies and annotation of *Slavum lentiscoides* and *Chaetogeoica ovagalla*.

Parameters	*S. lentiscoides*	*C. ovagalla*
NGS (Next-generation sequencing)		
Data (Gb)	58.13	36.40
Depth	184.11×	125.51×
Estimate genome (Mb)	293.69	276.95
Repeat sequence (%)	41.0	40.5
Heterozygosity (%)	0.99	0.70
Nanopore		
Clean data (Gb)	44.14	55.02
Depth	130.53×	185.62×
Assemble size (Mb)	336.27	293.08
Number of contigs	313	128
GC content (%)	32.50	32.08
Contigs N50 (Mb)	6.79	5.38
Hi-C		
Clean data (Gb)	40.35	45.54
Number of chromosomes	34	54
Unique mapped reads (%)	59.68	43.35
Chromosome anchored rate (%)	98.81	98.85
Chromosome anchored size (Mb)	332.26	289.72
Scaffold N50 (Mb)	19.77	11.85
Scaffold N90 (Mb)	11.05	8.38
Mean length (Mb)	1.66	4.25
Longest length (Mb)	46	17
Annotation		
Repeat sequence (%)	25.94	20.90
Number of predicted genes	11,747	14,492
Number of annotated genes	11,656 (99.22%)	13,004 (89.73%)
Average gene length (bp)	7,625	10,338
Average CDS length (bp)	208	1459
Average exon length (bp)	259	218
miRNA	27	31
rRNA	66	68
tRNA	200	172

Different assembly strategies were used to assemble the genomes of these two aphid species. For *S. lentiscoides*, the Nanopore long reads were initially cleaned using Canu v1.5^23^ for sequence error correction and were then assembled from the error-corrected reads with the parameters “useGrid = false, genomeSize = 300 m, minReadLength = 2000, minOverlapLength = 500, corOutCoverage = 135, corMinCoverage = 2”. We used the *Redundans*^24^ pipeline to remove bubble contigs from the draft assembly. For *C. ovagalla*, the long reads were analyzed using Nextdenovo v2.1^25^ for correction with the parameter “-seed_cutoff = 30k”, and then the corrected reads were assembled by SMARTdenovo (https://github.com/ruanjue/smartdenovo) with the parameters “-k 21 -J 5000 -t 20”. We found that the Canu assembly pipeline run obviously more slowly than the latter methods. These two draft aphid genome assemblies were subjected to two rounds of error correction using RACON^26^ with Nanopore reads, followed by three rounds of additional polishing using Pilon^27^ with Illumina sequencing data. We further removed a few contigs with microbial contamination by BLAST searches against the NCBI nt database, and no contigs related to the mitochondrial genome were found. After assembly and correction, we produced draft genome assemblies of *S. lentiscoides* and *C. ovagalla*, comprising a total of 336.27 Mb and 293.08 Mb of sequences, with 313 and 128 contigs, in which the contig N50 lengths were 6.79 Mb and 5.38 Mb, respectively (Table 1).

**Fig. 2:**
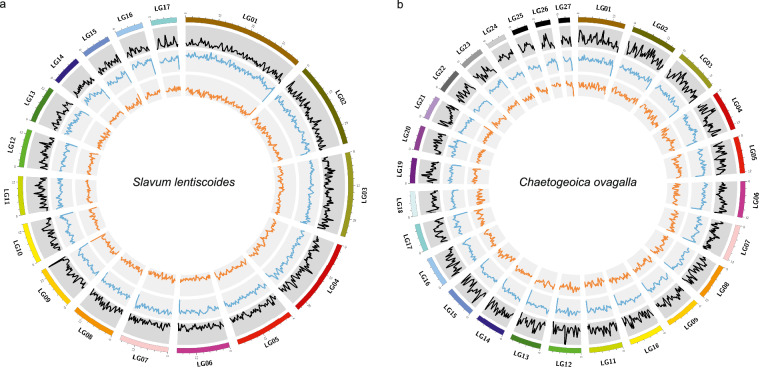
Circus plots describing genomic characteristics of *Slavum lentiscoides* (**a**) and *Chaetogeoica ovagalla* (**b**). From outer to inner circles: chromosome length, gene density, transposable element density, and GC density.

The Hi-C paired-end reads were mapped to the Nanopore assembly using BWA aln v0.7.10-r789^28^. The unique read pairs around the DpnII site were determined. Then, we applied LACHESIS software^29^ to cluster, order, and orient contigs from the draft assembly. Invalid read pairs were filtered with HiC-Pro^30^ using default settings. The predicted chromosomal genome was divided into bins of equal length (500 kb), and the number of valid Hi-C read pairs between bins was then used to represent the interaction signals between bins. Finally, we constructed a heatmap based on these interaction signals by R. For *C. ovagalla*, considering the variability in chromosome numbers observed in Aphididae^31^, we initially established a range of 10 to 30 for the cluster number. After careful consideration and analysis, we determined that a cluster number of 27 was the most appropriate choice. As a result, 40.35 Gb and 45.54 Gb of Hi-C clean reads were generated to build chromosome-level assemblies. After clustering, ordering, and orientation of the contigs, we obtained two final assemblies with chromosome-anchored sizes of 332.26 Mb and 289.72 Mb and scaffold N50 lengths of 19.77 Mb and 11.85 Mb for *S. lentiscoides* and *C. ovagalla*, respectively (Table 1). Therein, 128 and 86 contigs could be anchored to 17 and 27 linkage groups, with 98.81% and 98.85% anchoring rates, respectively (Table 1, Fig. 1e,f). The chromosome circus plots were generated using Circos v0.69-9^32^ (Fig. 2).

### Genome annotation

Before gene prediction, a de novo repeat library was built by using LTR_FINDER^33^ and RepeatScout^34^, and the library was classified by PASTEClassifier^35^. Transposable element sequences in the genome were predicted with RepeatMasker^36^ using the de novo library and Repbase library^37^. Consequently, we identified 87.71 Mb and 61.25 Mb of repeat sequences, accounting for 25.94% and 20.90% of the genome assemblies of *S. lentiscoides* and *C. ovagalla*, respectively (Table 1).

The protein-coding genes were predicted by integrating the evidence generated via the de novo, homology-based, and RNA-seq-based methods. For ab initio prediction, we used Augustus v2.4^38^ to generate the de novo predictions with the default parameters. For the homology-based analysis, GeMoMa v1.3.1^39^ was used to query the protein sequences against a database of four species (*Myzus persicae*, *Aphis gossypii*, *Rhopalosiphum maidis*, and *Acyrthosiphon pisum*). For RNA-seq annotation, RNA-seq reads were aligned to the genome assembly using HISAT2 v2.0.4^40^ and StringTie v1.2.3^41^ with the default parameters. TransDecoder v2.0^42^, GeneMarkS-T v5.1^43^, and PASA v2.0.2^44^ were used to generate transcript predictions. Finally, we integrated three types of evidence for gene prediction by EVidenceModeler (EVM) v 1.1.1^45^. By integrating *ab initio*-based, homology-based, and transcriptome-based evidence, we predicted 11,747 genes in the *S. lentiscoides* genome, and 14,492 genes in the genome of *C. ovagalla*. The predicted genes were then searched for homology-based functions by BLAST searches against the NCBI non-redundant protein sequences (nr), Eukaryotic Orthologous Groups (KOG), TrEMBL, Kyoto Encyclopedia of Genes and Genomes (KEGG), and the Gene Ontology (GO) databases with BLASTP v2.2.31^46^ (-evalue 1e-5). The GO terms were assigned using blast2go v5.0^47^. A total of 99.22% (11,656 genes) and 89.73% (13,004 genes) of the predicted genes of these two genomes were supported by functional annotation from the above five protein databases. Among these genes of *S. lentiscoides* and *C. ovagalla*, 98.11% and 89.15% showed homology to proteins in NCBI nr, 98.89% and 67.84% in TrEMBL, 67.34% and 57.00% in KOG, 48.76% and 43.27% in KEGG, and 38.11% and 43.17% in GO, respectively (Table 2).

**Table 2:** Number of functionally annotated genes of *Slavum lentiscoides* and *Chaetogeoica ovagalla* genomes.

Annotation database	*S. lentiscoides*		*C. ovagalla*	
	Gene number	Percentage (%)	Gene number	Percentage (%)
GO	4,477	38.11	6,256	43.17
KEGG	5,728	48.76	6,271	43.27
KOG	7,910	67.34	8,260	57.00
TrEMBL	11,617	98.89	9,831	67.84
NCBI nr	11,525	98.11	12,919	89.15
Total annotated	11,656	99.22	13,004	89.73

Three types of noncoding RNAs (ncRNAs) were identified. The microRNA (miRNA) and ribosomal RNA (rRNA) genes were predicted by BLASTN searches against the Rfam database^48^. The transfer RNA (tRNA) genes were identified using tRNAscan-SE^49^. Finally, we identified 27 and 31 miRNAs, 66 and 68 rRNAs, and 200 and 172 tRNAs within the genomes of *S. lentiscoides* and *C. ovagalla*, respectively (Table 1).

## Data Records

The genome raw data, RNA sequencing data, and genome assemblies are available at the National Center for Biotechnology Information (NCBI) under the BioProject accession numbers PRJNA765394 and PRJNA832539. The Illumina WGS data was archived with the accession numbers SRR16046963^50^ and SRR23999325^51^. The Nanopore WGS data was deposited with the accession numbers SRR16046964^52^ and SRR23999326^53^. The RNA-Seq data was archived with the accession numbers SRR16046961^54^ and SRR23999323^55^. The Hi-C data was archived under the accession numbers SRR16046962^56^ and SRR23999324^57^. The genome assemblies have been deposited at GenBank under the accession numbers GCA_032441835.1^58^ and GCA_032441825.1^59^. The genome annotation files have been deposited at the Figshare^60^.

## Technical Validation

The quality and completeness of draft assemblies were assessed by three methods as follows. The Illumina reads were mapped to the draft assembly with BWA-MEM v0.7.10-r789^61^, and the mapping ratio was calculated with SAMTOOLS v1.3^62^. In addition, the Core Eukaryotic Genes Mapping Approach (CEGMA v2.5^63^) and BUSCO v3.1.0^64^ were used to assess the completeness of the genome assembly.

The *S. lentiscoides* and *C. ovagalla* genomes contained 96.77% and 97.58% of highly conserved Core Eukaryotic Genes (CEGs), respectively. They showed a high representation of conserved complete Benchmarking Universal Single-Copy Orthologs (BUSCOs) (*S. lentiscoides*: 93.70% complete BUSCOs of insecta_odb10; *C. ovagalla*: 97.00% complete BUSCOs of insecta_odb10), mapped with 96.78% and 97.19% of Illumina short reads, respectively, which indicated that these genome assemblies were of high quality and near-complete.

### Supplementary information

Figure S1

## Data Availability

This paper does not report original code. If no detailed parameters were mentioned for the software, default parameters were used according to the software introduction.
