# Supplementary figures and images for: Two chromosome-level genome assemblies of galling aphids Slavum lentiscoides and Chaetogeoica ovagalla
Source: Sci Data. 2024 Jul 20;11:803. doi: 10.1038/s41597-024-03653-x (PMC11271456; doi:10.1038/s41597-024-03653-x)

**
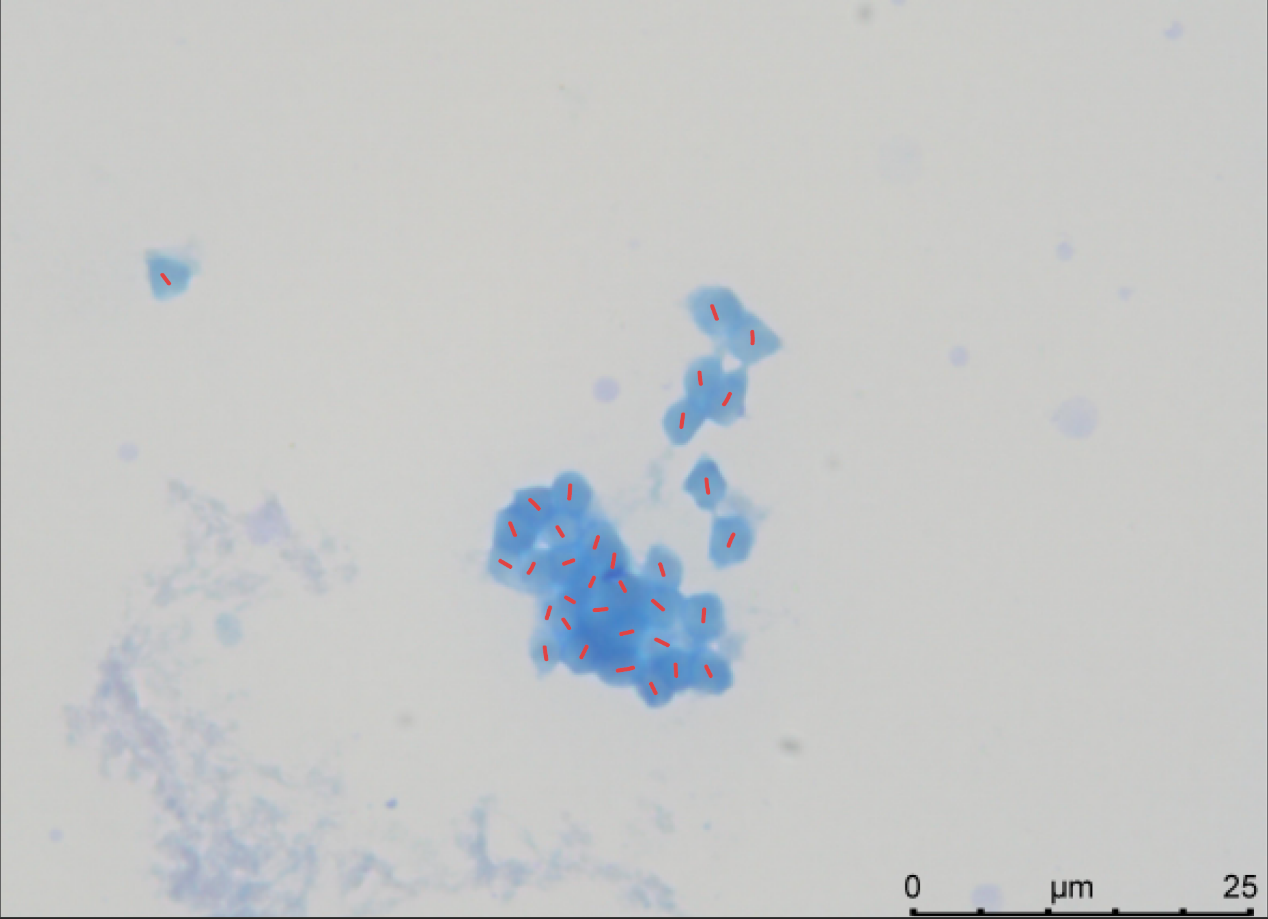
**

**Fig. S1.** The chromosome karyotype of *Slavum lentiscoides* determined by Giemsa staining.

Supplement: Supplementary file 1 — Figure S1 [file 41597_2024_3653_MOESM1_ESM.docx]
